# Identifying Thematics in a Brain-Computer Interface Research

**DOI:** 10.1155/2023/2793211

**Published:** 2023-01-04

**Authors:** Hadeel Alharbi

**Affiliations:** Department of Information and Computer Science, College of Computer Science and Engineering, University of Ha'il, Ha'il 81481, Saudi Arabia

## Abstract

This umbrella review is motivated to understand the shift in research themes on brain-computer interfacing (BCI) and it determined that a shift away from themes that focus on medical advancement and system development to applications that included education, marketing, gaming, safety, and security has occurred. The background of this review examined aspects of BCI categorisation, neuroimaging methods, brain control signal classification, applications, and ethics. The specific area of BCI software and hardware development was not examined. A search using One Search was undertaken and 92 BCI reviews were selected for inclusion. Publication demographics indicate the average number of authors on review papers considered was 4.2 ± 1.8. The results also indicate a rapid increase in the number of BCI reviews from 2003, with only three reviews before that period, two in 1972, and one in 1996. While BCI authors were predominantly Euro-American in early reviews, this shifted to a more global authorship, which China dominated by 2020–2022. The review revealed six disciplines associated with BCI systems: life sciences and biomedicine (*n* = 42), neurosciences and neurology (*n* = 35), and rehabilitation (*n* = 20); (2) the second domain centred on the theme of functionality: computer science (*n* = 20), engineering (*n* = 28) and technology (*n* = 38). There was a thematic shift from understanding brain function and modes of interfacing BCI systems to more applied research novel areas of research-identified surround artificial intelligence, including machine learning, pre-processing, and deep learning. As BCI systems become more invasive in the lives of “normal” individuals, it is expected that there will be a refocus and thematic shift towards increased research into ethical issues and the need for legal oversight in BCI application.

## 1. Introduction

Advances in technology have given hope to many individuals who suffer from chronic brain-related medical conditions [1]. Those with affective brain disorders have been given hope that a level of disability caused by their conditions can be alleviated, and they can enjoy a more “normal” life [2, 3]. While popular culture has created images of the ultimate cyborg, this technology is far from becoming a reality, and while there have been major advances, there is still a long road before human and machine are fully integrated. However, the first step on this path is underway with advances in brain-computer Interfacing.

Brain-computer interface (BCI) is a complete system of software and hardware that facilities the direct communication path between the brain and a device, which enables that device to be controlled with the issuance of commands or external interventions [4, 5]. Functionally, BCI involve two components: the user and the computer that enter reciprocal interactions [6]. The key to the effective operation of BCI systems is the robust filtering of signals to enable effective and continuous feedback to the user [6]. As an enhanced understanding of the brain is achieved, there will a generation with further advances in BCI performance as task-specific activity patterns and their ability to become accurately detected, their features comprehended, optimized, and classified [7–9]. This intrinsically ties to the development of software systems linked to artificial intelligence (AI), including machine learning (ML), pre-processing, and deep learning (DL) that will improve flexibility, extendibility, usability, and performance at the individual user level [10–18].

The field of BCI is rapidly developing; in 2006, people with paralysis were moving computer mice, and by 2012, this has advanced to the control of robot arms [19, 20]. While the development of BCI products has primarily been driven by medical research fields, these developments have a potential crossover potential into the education, gaming and entertainment, marketing and advertising, military, and safety and security sectors [5, 20, 21]. These developments have led to improvements in the quality of life of individuals, particularly those living with chronic disability and those that care for them [22]. The development and advances in BCI technology is being driven by new players with the involvement of the nonmedical tech billionaires and their ability to fund research at almost unlimited rates [23].

There has been a shift in the research paradigms in the BCI field and this review seeks to understand those shifts in terms of thematic changes in the literature [24–29]. At present, there is little understanding of how the themes in the literature have changed through time. Due to the increasing volume of literature surrounding BCI, the optimal method to obtain an overview of the thematic changes through time was via an umbrella review. This review first outlines the research objectives and questions that are the focus of the work. A background review into aspects of BCI categorization, neuroimaging methods, brain control signal classification, applications, and ethics is present. This is followed by the thematic review.

### 1.1. Objectives and Research Questions

This umbrella review aims to provide a brief overview of BCI systems, examining aspects of categorization, neuroimaging methods, brain control signal classification, its applications, and ethics all of which are contained within the background of this paper and used to inform on the thematic analysis which is undertaken after the overview. This review does not explore BCI hardware or software. This review also aims to examine the demographics of the BCI reviews, and these publications then explored to gain a comprehensive insight into the current BCI research themes.

To satisfy these aims, this review asks three questions:

  Question 1: How are BCI systems categorized and applied (background)?
  Question 2: What are the demographics metrics for BCI review publications?
  Question 3: What are the BCI research foci as reflected by the thematics of review publications through time?

## 2. Background

How BCI systems are developed and implemented is highly dependent on the target user. Medical use of BCI systems has two aspects, diagnostics and rehabilitation, and has been shown to have beneficial in monitoring and improvement in the quality of life for a variety of conditions [30]. Early work in BCI systems was technologically limited to brain signals using electroencephalography and limited applications neuropsychology and neurophysiology and understanding brain regionalization and functioning, and of the potential for use in motor support and medical diagnostics [31]. However, the development electrocorticography has provided more accurate and long-term brain signal applications not just in the medical field. Furthermore, the use of metabolic signals with functional magnetic resonance imaging and other systems provides more avenues to work with areas of the brain that have been damaged and are unable to emit lucid signals.

### 2.1. BCI Categorization

These three aspects to the classification of BCIs revolve around aspects of dependability, invasiveness, and synchronization [4]. Dependability is determined by the level of motor control the individual needs to interact with the BCI. Invasiveness reflects how the BCI is deployed to capture brain signals. Synchronization reflects the time periodicity that the individual interacts with the BCI system. Subclassification of BCI systems can reflect aspects of targeted subjects, such as animals or humans, and how signals are transmitted in the system, wireless or fixed line [30].

#### 2.1.1. Dependability

The level of dependability is determined by the level of motor control that is required by the individual [4]. Dependant BCIs assist the individual to undertake takes more easily, such as gaming or using mobility devices. Independent BCIs do not require external controls and rely completely on brain signal detection.

#### 2.1.2. Invasiveness

There are three forms of BCI invasiveness based on the position of electrodes [32–34]: (1) invasive BCIs that are embedded into the brain; (2) partial invasive BCIs that have the device implanted inside the skull but outside the brain; and (3) non-invasive BCI systems uses neuron imaging outside the skull. Invasive BCIs involve the implanting of microelectrodes that are implanted in the brain [4, 35]. While invasive methods provide high-quality signaling, this is temporary as the buildup of scar tissues through time reduces the signal quality [5]. Once these microelectrodes have been implanted, they are fixed and cannot be used to monitor other parts of the brain [4]. Semi-invasive BCI systems are located under the skull but are not attached to the brain [5]. Like invasive BCI systems, semi-invasive systems remain in place and can only monitor one area of the brain. Noninvasive BCIs form the basis for most applications and involve recoded signals on the scalp [4]. While the signal quality is not as high with more invasive approaches, noninvasive methods do not require surgery [4].

#### 2.1.3. Synchronization

The level of synchronization is determined by the periodicity that the individual interacts with the BCI system [4]. Synchronous BCI systems only operate during periods when the individual chooses to use them [36]. Synchronous systems allow for the use of motor actions, such as blinks, when brain signals cannot be analyzed [37]. In contrast, asynchronous, or self-paced, BCI systems enable the individual to undertake tasks at any time and are always functionally active [4]. Asynchronous systems do not require external cue stimuli and have natural modes of interaction making them more user-friendly [36, 37]. Advances in asynchronous systems have seen increased levels of autonomy and empowerment of BCI users with the development of brain switches that enable the user to disengage with the BCI system [38].

### 2.2. BCI Neuroimaging

Understanding areas of the brain and their function enables targeted BCI to be developed that aim to detect specific signals these may be electric or metabolic [4, 5, 27, 39–42]. Electric signals fall into the bands: alpha (8–12 Hz) which is associated with closing the eyes and relaxed states, awareness without concentration, good mood, calmness, learning new information, and self-awareness; beta (12–30 Hz) are emitted with energetic thinking, attention and alertness, and anxiousness; gamma (25–100 Hz) which associates with writing and reading; delta (1–4 Hz) related to deep sleep and unconsciousness; mu (7–13 Hz) that deals with motor neurons in a rest state; and theta (4–7 Hz) that are associated with sleep [37, 40, 43, 44]. As the understanding of brain function has improved, it has become evident that different areas of the brain have the potential to affect movement, where an individual controls motor monument involves a different region of the brain to where the same imagined movement is occurring [45]. The technologies that assisted motor activities required invasive systems, such as connection to the nervous system [46].

#### 2.2.1. Electrocorticography (ECoG)

ECoG is a portable semi-invasive system where electrodes are placed between the brain and the skull. There are two forms of ECoG, monitoring under surgical conditions and long-term implants. One of the major benefits of the ECoG is the recoding of multiple sensorimotor rhythms that operate at different frequencies at the same time [37, 43, 44]. ECoG is advantageous due to higher levels of spatial (∼1 mm) and temporal resolution (∼0.003 s) [4, 37, 47]. Historically, ECoG systems were used under surgical conditions that benefit short-term monitoring [4]. Due the restricted time in surgery that data are being collected is possible that periodic irregularity such epilepsy, and due to this irregularity, these may not be picked up during the procedure [4].

Apart from short-term monitoring, the use of implanted semi-invasive systems has been the long-term use of ECoG. Cortical electrical stimulation (CES) systems have been investigated for a variety of motor functions such as hand movement [35, 48] and for nonmotor applications to enhance auditory visual and language systems has been the focus of research [49]. ECoGs provide long term constant and stable signal acquisition systems and therefore have the potential for addressing chronic conditions [48]. However, there are significant risks with the use ECoGs due to their invasive nature; these risks include epi- and subdural hematoma, cerebral infarcts, increase of intracranial pressure, and brain edema [28, 47, 49, 50].

#### 2.2.2. Electroencephalography (EEG)

EEG is a portable noninvasive monitor of the neuron electrical activity of the brain with the use of electrodes that are attached to the scalp [4, 5, 51]. Attached electrodes are used to detect and capture signals that are emitted from the brain. There are two forms of electrode: active electrodes, which contain an amplifier; and passive electrodes, which do not contain an amplifier [4, 5]. EEGs have significant benefits that provide most of the required information for the brain, the system is inexpensive, it has no adverse side effects, and there is no need for using external electrical signals or voltage [4, 43]. However, EEG systems have the spatial resolution (∼10–30 mm), the lowest of all neuroimaging techniques, while temporal resolution is quicker (∼0.05 s) [37, 43, 50, 52]. Advances in emotion detection such as levels of meditation, engagement, frustration, excitement, and stress are examples of affective and cognitive feedback and regulation using EEG [53]. EEG has two applications: (1) in medicine with the provision of enhanced monitoring, assessment, and diagnosis of psychiatric and neurological disorders such as autism, depression, and schizophrenia [54] and (2) in entertainment, design of traffic safety systems and gaming through understanding emotional feedback assisting in product design and development [54]. Research has focused on the level of individual variability and reducing can lead to long tedious calibration times to ensure task determination accuracy [55].

#### 2.2.3. Functional Magnetic Resonance Imaging (fMRI)

fMRI is a nonportable noninvasive method that maps the shifts in task-induced blood oxygen levels within the brain that are associated with different neural activities [4, 5, 39]. The benefits of fMRI are that it produces high spatial resolution (∼1 mm), but this method has a slight time lag (∼1 s), which means it is very sensitive to movements and it is also noisy and has a high cost [4, 37, 43, 50]. fMRI can be used to provide real-time whole brain analysis which allows individuals to self-regulate specific brain regions to control external devices [39, 43]. The individual control is enhanced through time with multivoxel pattern analysis, a system of machine learning that improves individual control and improves the filtering of interference through time [39].

#### 2.2.4. Intracortical Implants

Intracortical implants are invasive and attached directly to targeted areas of the brain. They have high spatial resolution (0.05–0.5) and fast temporal resolution [37]. These implants carry high risks of infection and biological rejection and have long term signal loss [49, 56]. Early systems to enhance vision used sixty-eight electrodes implanted on the surface of the brain to enable improved vision [46]. The use of intracortical BCI systems were a major area of research using animals in the early days of BCI development, but these never fully reached their potential in human trials, and this research was gradually replaced with less invasive techniques [32, 57]. The use of invasive devices also has ethical problems with questions such as reversibility, with the brain returning to its normal state after the BCI is removed, practically if there has been a buildup of scar tissues [22].

#### 2.2.5. Magnetoencephalography (MEG)

MEG is a noninvasive nonportable BCI that relies on the detection of changes in the magnetic fields on the surface of the scalp using superconducting quantum interference devices (SQUIDs) [4, 5]. These changes in magnetic fields are a consequence of neuron electrical activity. The benefits of MEG include moderate spatial (∼0.05 mm) and temporal resolution (∼0.05 s) [37]. The magnetic fields utilized by MEG are less influenced by electric currents; however, the equipment is very expensive and loses resolution at the surface and deep in the brain [4, 43].

#### 2.2.6. Near-Infrared Spectroscopy (fNIRS)

fNIRS is portable noninvasive and relies on projecting infrared light into the brain to measure changes in wavelength caused by changes in blood oxygenation [5]. While fNIRS is limited in temporal resolution (∼1 s) and provides medium spatial resolution (∼5 mm), it is able to provide real-time information on the different brain activation patterns associated with tasks and their difficulty [4, 37, 43, 50, 58]. However, external device speed is limited by the detection of delayed metabolic response speeds to activity, with up to 16 s needed for a list selection from a single channel [43]. Benefits include low cost compared to other imaging systems and did not require long-term training by the BCI user [43]. Optimal use of fNRIS has been achieved with the primary moor cortex and prefrontal cortex with applications for motor tasks, and cognitive tasks such as arithmetic, music imagery, and emotion induction [59].

#### 2.2.7. Positron Emission Tomography (PET)

PET is a nonportable noninvasive approach that used a metabolic process using gamma rays that are generated by the interaction of radionuclide-emitted positrons and elections [4]. PET is expensive and does not provide images or cross-sections of the brain and therefore does not show the location of physical abnormalities. The PET method allows for the detection of most brain activity.

#### 2.2.8. Single Photon Emission Computed Tomography (SPECT)

SPECT nuclear tomographic imaging is based on gamma rays that are emitted by radionucleotides that have been injected into the blood stream [4]. Like PET, SPECT is expensive, but with the use of brain tissue-specific binding chemicals, it provides 3D images of the monitored area of the brain [4].

### 2.3. Brain Control Signal Classification

There are three brain control signals that are utilized by BCI systems [4]: (1) visual evoked signals are those generated unconsciously by the individual in response to external stimuli; (2) spontaneous signals are those generated by the individual voluntarily without any external stimuli; and (3) hybrid signals are a combination of signals that are used for control.

#### 2.3.1. Evoked Signals (VEP)

There are two forms of VEP. First, steady-state evoked potentials (SSEP) are triggered by signal modulation in the visual cortex when the individual receives periodic stimulus such as a moving image, modulated sound, or vibrations; the individual can elicit a response that can be task specific such as controlling a button stick [4]. SSEP has an information transfer rate of 60–100 bits/min [37]. Second, P300 is an EEG signal that is detected positive peaks when the individual is exposed to an infrequent task, while this does not require training, the triggering required needs respective stimuli that can be tiring [4]. P300 has an information transfer rate of 20–25 bits/min [37].

#### 2.3.2. Spontaneous Signals

There are three spontaneous signals that are used in BCI systems. First, motor and sensorimotor rhythms that are associated with motor actions such as the movement of limbs and are derived from the motor cortex. Individuals through operant conditioning can be trained to voluntarily change the amplitude of their sensorimotor rhythms to signal processes [4]. Alternatively, motor intention can be detected via EEG which can then control devices such as a computer mouse or play a computer game [4]. Second, slow cortical potentials (SCP) detect nonmotor signals from the frontal parts of the brain, and after training, an individual can learn to control the generation of signals that can be translated into motor tasks, with benefits for those with motor cortex damage [4]. Third, the use of nonmotor cognitive task signals can be used to perform music imagination, visual counting, mental rotation, and mathematical computation [4].

#### 2.3.3. Hybrid Systems (hBCI)

There has been an increasing rate of research into the use of integrated systems that involve more than one BCI system; these are known collectively as hybrid systems [44]. hBCI systems utilize a combination of at least one brain-generated signal with another neurological, physiological, or external signal to increase reliability and individual control [4, 60, 61]. There are six types of hBCI that are in use [62, 63]; Liu et al.: (1) two different EEG BCIs; (2) EEG and a non-EEG BCI; (3) EEG BCI and another biosignal; (4) EEG BCI and EEG-based monitoring; (5) EEG BCI and other signals; and (6) EEG BCI, EEG monitoring, and other biosignals. There are three classifications of hybrid BCI systems [60]: (1) the source of brain signals, (2) the form of brain signals, and (3) the operation of the system. There are many real-world applications for hBCI systems such as detecting awareness, gaming, mouse control, navigation, neuroprosthetics, and wheelchair operation most of which rely of visual modalities, while others require some degree of physical movement [60, 61]. hBCI has a higher classification accuracy and information transference, with lower false positives and artifact detection, leading to an increased response and improved rehabilitation outcomes [62, 64]. Furthermore, hybrid systems have the potential to enable individuals to perform different tasks at the same time, but this may lead to dual-task interference [62, 65]. Hybrid systems are more difficult to operate due to the complexity of users and performance can decline for some who shift from a single BCI system [65].

### 2.4. BCI Applications

Historically, the focus of early BCI systems was to provide alternative output pathways for severely disabled individuals that would enable them to control external systems [33, 45]. However, one of the main challenges for researchers in BCI system application is the training of individuals to use control mechanisms, and there are related to habituation and response rates, the actual time taken to train the user and fatigue, the need to modulate each device to the needs of the individual, lack of predictive indicators of performance due to individual circumstances, limited applicability, the ability to control the desired task with differences in system activity, and the self-pace requirements of the individual [66–68]. While BCI systems offer hope to many, there is a demographic who are BCI illiterate, with illiteracy rated ranging from 48.7–61.6%, and for these individuals, the promises made by researchers can lead to frustration and disillusionment [66, 69, 70]. Notwithstanding, BCI integration with such devices has occurred with a wide range of applications such as prosthetics and exoskeletons, robots, spelling and communication systems, cursors, joysticks, and wheelchairs [71, 72].

#### 2.4.1. Education

BCI systems have been developed to assist in the understanding of student performance, particularly those with neurodevelopmental disorders [21, 73, 74]. The use of EEGs in educational setting allows teachers to gauge the level of engagement of the learner, enabling an effective design of educational tools maximizing focused learning [54, 73]. Similarly, self-regulation and skill learning using through monitoring brain signals using fMRI enable the individual to determine the level brain regional focus [5].

#### 2.4.2. Gaming and Entertainment

Gaming has become an avenue for BCI research and applications [25, 73]. While simplified games have been developed to assist in training, the commercial gaming using BCI systems is primarily for “healthy” individuals [25]. The use of BCI for gaming has led to the development of software enabling individuals to fly virtual aircraft or play multiplayer online games, where hBCI systems have been used to create driving simulators and flight control systems [60]. *Brain Arena* is a football game where collaborative gaming between players using individual BCIs control the ball by imaging left or righthand movements [5]. More recently, classic games have been converted for BCI systems “Bacteria Hunt,” “Mind Balance,” “Pacman,” “Pinball,” “Tetris,” and “World of Warcraft” [75]. Furthermore, EEG data can be also used to observe relationships between multimedia experiences and the human emotions. Designers use the information obtained in relation to the affect stated of the user to enhance the experience through the moderation of difficulty, punishment, and reward systems [54].

#### 2.4.3. Marketing and Advertising

Marketers have utilized BCI to monitor individual repones to advertise commercial products and political campaigns. In particular, an assessment is made of individual attention generation that is associated with watching activity [5]. The use of EEG to match neurophysiological parameters with potential customer responses to stimuli enables the effective design of campaigns [54].

#### 2.4.4. Medical

Medical interventions have remained the main research focus that has driven much of the development of BCI innovations [73]. These applications have delivered real-world benefits for diagnostics and for rehabilitation for those with cognitive impairment of movement and function control [59, 76, 77]. Medical applications rely on adaptive neural plasticity where damaged areas of the brain are bypassed [78, 79]. While some BCI systems training is for prolonged use, training in the short-term helps navigate brain blockages and enhances motor function and assists in brain recovery [80, 81].

The use of noninvasive BCI is well-established in medical diagnostics. The medial use of EEG to detect brain diseases, epilepsy, disorders, coma, encephalopathies, and brain death but does not provide information on the location of injuries or physical abnormalities like tumors, as well as offer monitoring of specific behaviors [4, 54, 82]. EEG has also been used to detect mental commands for the operation of wheelchairs after spinal cord injury or neuromuscular disease and has extensively been used in stroke rehabilitation and external device control [35, 43]. EEG systems in stroke victims have been effective in returning upper body control after rehabilitation [76, 83–86].

Other than EEG, noninvasive BCI systems do not receive as much attention in the literature. MEG provides a temporal and spatial resolution of the brain, enabling the detection of regions causing epilepsy or tumor or other mass lesions [4]. PET imaging while not providing the location of the injury is able to effectively detect abnormities making it useful for discerning the presence of absence of brain diseases, disorders, coma, encephalopathies, and brain death [4]. The use of infrared thermography to detect changes in uses deep breathing, blinking, or opening of the mouth as device commands has been developed that allows for the movement of wheelchairs [43].

hBCI systems are becoming more frequent in medical interventions [61]. Honda's brain-machine interface uses a hBCI model headset containing EEG and NIRS sensors set to control a robot where the individual uses thoughts which then are translated into motor actions [5]. Other hBCI systems bring added mobility with wheelchair operation, neuroprosthetics, and mouse control [61].

Communication and BCI systems have become a major area of research [77, 87]. There are significant benefits to spelling and writing systems, and with commercialization, such as “Flash Type” and “Bremen Speller”; they are user friendly with mostly not requiring specific training [87–89]. However, small levels of interference disrupt the system and cause errors, particularly if the system has been compromised, making security arouse BCI systems imperative [90].

Semi-invasive closed-loop ECoG implants are well-known medical interventions. Cochlear implants are a major commercial use of BCI to restore hearing to the deaf, enabling hearing and subsequent speech and language skill development [5, 43]. While bionic eyes are rapidly being developed in the form of a small digital camera, external processor and an implant with a microchip and stimulating electrodes surgically placed in the back of the eye [5, 43]. Similarly, ECoG technology where evoked somatosensory perception is being developed for motor neuroprosthetics aims to bring mobility and functional ability to those individuals with paralysis [5, 49].

#### 2.4.5. Security and Safety

BCI systems are currently under development to assist road user safety, as well as security and military applications [5, 21, 22]. The detection of driver fatigue and alertness has a major implication for road safety and is a cutting-edge area of research in the application of BCI [5, 73]. The technology utilizes the recognition of cyclic sleep patterns and eye movement to detect changes in driver eye behavior. hBCI systems use visual cues and physical movements to detect awareness in drivers [60]. The use of BCI enhancement systems and its applications of enhancement and transhumanism is a research area that the military has increased interest in [22]. fMRI application can be used to operate robots through a visual perspective [43]. Additionally, the use of BCI systems to enable the control of building systems in smart cities has been studied [91, 92]. The military can use the BCI system to monitor troops during operations using cost-effective portable sensors; these could alert to changes in the mental capacity of the troops under observation [21].

### 2.5. BCI Ethics

The literature on BCI is dominated with the application and processes surrounding BCI systems; little has been done to review the ethical implications, and this raised more questions than answers [22, 93]. Historically, ethics were not a focus in reviews, although the procedures are more invasive than modern to BCI systems [32, 46]. The most discussed ethical issues that surround BCI are user safety and risk benefits associated with their use, particularly those surrounding the use of invasive and semi-invasive technologies [22, 73, 94]. However, there are three areas in need of ethical consideration when dealing with BCI applications and their users: (1) The ability of an individual that utilizes a BCI system to provide informed consent and their loss of personhood. (2) The use of machine learning and the self-empowerment of BCI systems and the abrogating responsibility by the user of these systems. (3) Issues that deal with researcher responsibility. Each of these areas presents ethical dilemmas in terms of agency, self-image, identity, and responsibility for any BCI system applications [20]. Although more recently data management and its privacy has become an increasing concern [73].

#### 2.5.1. Humanity, Personhood, and Consent

Informed consent is a major concern ethically in BCI research. There is a nuanced difference between the individual giving assent to perform experimentation or implantation of a system, and the individual has the ability to give consent [22]. Informed consent has three components that need to be satisfied [22]: (1) the individual can understand all the disclosure information; (2) the individual has the capacity to make a rational and reasonable decision based on that information; and (3) that the decision is to totally voluntary and made without coercion or influence. The ability to provide informed consent is a major concern when the patients are in locked-in or noncommunicative states, particularly if these individuals do not want to participate in BCI programs [22]. There is often a pressure to conform to what is medically considered the “normal” body or form of life and whether is it socially acceptable to live with a perceived deficit [22]. For instance, with the use of cochlear implants, some individuals do not see themselves in terms of having a deficit, thus implants may not be seen as treatments but rather enhancements [22]. Such examples highlight the differences between individuals in perception; this is ethically critical to understand how personhood and humanity are negotiated and consent is negotiated and obtained.

#### 2.5.2. Loss of or Inappropriate Control

Ethics surrounding the use of BCI systems is often overlooked, particularly as these systems are growing in their autonomous capabilities, particularly as machine learning empowers individuals with major motor disorders; there are three major areas of ethical consideration that need to be addressed in terms of individual use of a BCI system [22]: (1) If systems become self-empowering with machine learning, at what point to the systems inhibit the individuals ability to act on their own desires, and thus, those individuals lose control of their thoughts and behaviors depriving them of agency? (2) As BCI systems become more sophisticated and semiautonomous, who is responsible if the system enables the user to perform an action that is morally or legally inappropriate, and does the use of that system impede the ascription of responsibility? (3) How are BCI system applications in terms of enhancement of neural pathways regulated in terms of military or religious applications?

#### 2.5.3. Researcher Responsibility

Researcher responsibility is less prominent in ethical considerations. The discovery of novel incidental findings that was not within a project's ethical scope can have implications for an individual's personal information and privacy [22, 66]. Consent obtained by the researcher for one project may have implications for other areas of research and the individual, therefore, may be “unaware of the extent of information that is being obtained from his or her brain” [22]. In interdisciplinary teams, how and what information is relevant to a researcher given their disciplinary area and should all information be shared within the team is a major ethical dilemma of integrative research [22]. When researchers report findings, how much consideration should be given to consulting the individuals that were the subject of that research, and how are issues where the individuals do not want those findings reported dealt with [22, 66]. Researchers also must also not overplay the expectations of application for individuals and their caregivers, overstating the benefits can often lead to disappointment and frustration [66]. Failure to address these issues leads to the dehumanizing of the research subjects and therefore is a serious moral concern, and there is an increasing call for the development of a code of ethical conduct for BCI systems [66].

### 2.6. Summary

Areas of applied BCI research can be divided into three areas: dependability, invasiveness, and synchronization, and advances in each of these areas have driven advances in all aspects of BCI systems. Neuroimaging has seen a shift from invasive methods toward semi and non-invasive technologies. Current technological advancement is seeing a gradual shift away from the dependency on electrical signals systems to those using metabolic signals to provide detailed information on brain function, and how best to provide medical care or assisted augmentation. As systems and technologies have improved in the detection and understanding of brain functioning, the use of single evoked spontaneous signals to greater use of integrated hybrid signal BCI systems. The growth in technological capability and diversity of BCI systems has led to increased real-world applications that have enhances a broad spectrum of human activity. While historically the focus on BCI applications has been dominated by the medical sector, more recently education, gaming, marketing and safety and security have all benefited from BCI system development. Ethically, there are many questions surrounding the use of BCI systems, and these reflect more than the safety and consent considerations but also deal with aspects of humanity and responsibility.

## 3. Methods

### 3.1. Search Strategy

This review uses *One Search*, a data mining tool that accesses 252 databases including BioOne, Google Scholar, JSTOR, ProQuest, and PubMed. The search criteria for this study included the keywords: “computer,” “brain,” “interface,” and “review,” all of which needed to be in the domains of title, abstract, or keywords. All forms of the review were included to maximize the discovery of themes, their primary word associations, and disciplinary determination.

### 3.2. Inclusion and Exclusion Criteria

**Figure 1:**
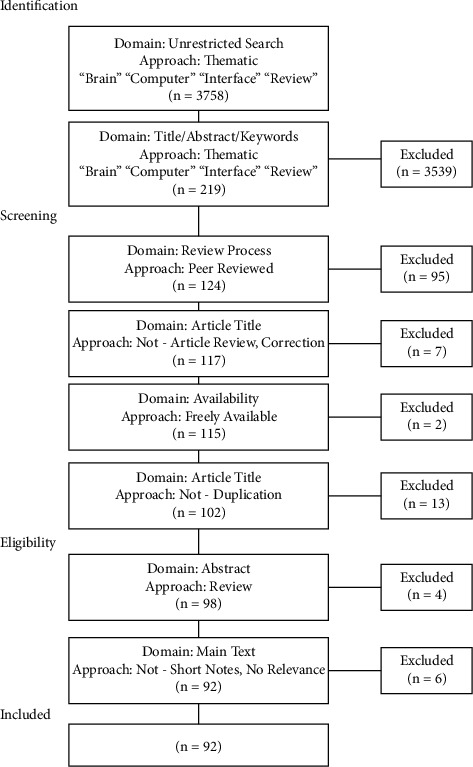
Flowchart of the BCI review article selection process.

Only reviews that were peer-reviewed were included for consideration (*n* = 124). No date ranges were applied to the search to enable the study of thematic shifts in research through time. Those papers that were not institutionally available at the time of the search were excluded (*n* = 2). Correction papers were omitted (*n* = 6), as were short research notes (pages <2; *n* = 2) and article reviews (*n* = 1). Repeats were removed (*n* = 13) along with articles that were not review articles (*n* = 4). Four articles were found to lack relevance: a glossary of terms (*n* = 1); law-related paper (*n* = 1); and interviews (*n* = 2). After initial exclusion, a total of 92 papers were retained and critically appraised [95]. Many early works (e.g. [46]) did not meet the modern review standard, missing methodology or clear statements of review questions; however, these were retained as they enable a more thorough understanding of the early literature and foci of the research at the time they were written. All modern papers pass the appraisal, a reflection in the shift in editorial standards and improved methodical requirements for publishing a literature review (Figure 1).

### 3.3. Publication Demographics

A bar chart of publications used in this review by year was produced to indicate any trend in the rates of publication. The number of authors per paper/year was noted and the overall mean was calculated (±SD). Author affiliations were mapped using worldchart.net to indicate shifts in researcher geographical domains. When authors have more than one country institution affiliation, it was counted for each country. Same country affiliation was only counted once for that country. Furthermore, publications returned using the unfiltered peer-reviewed *One Search* of “Brain-Computer Interface” (*n* = 8184) were charted to show changes in the number of publications per year through time. The *One Search - Subject Filter* results were also used to determine key disciplines that relate to the review papers, reviews may be interdisciplinary and thus counted more than once. Nondisciplinary object words such as “Brain” and “EEG” were omitted, and where duplications in disciplines occurred the largest value was used, such as the use of“neurosciences and neurology” resultand the omission of “neurosciences.”

### 3.4. Thematic Analysis

Articles were loaded into NVivo 12 Plus to identify work frequencies and shifts in word correlations. The 100 most frequently used terms were shown in the generated word cloud where articles were grouped into two cohorts that were designed to show shifts in word frequency and form a historical (pre-2018) period to more modern approaches (2018 onwards). The relative size to other words, words with higher usage are larger than those whose use is infrequent. As well as the NVivo default word omission, articles, pronouns, prepositions, and irregular verbs were removed; this study also removed noninformative terms such as “DOI,” numbers, and dates. A word-association frequency diagram constructed using the Pearson correlation coefficients was produced that showed relative word use and their spatial positioning between the two time periods using the 50 most frequent words for each time. Keywords were tallied and discussed in relation to the NVivo results.

## 4. Results and Discussion

### 4.1. Publication Demographics

**Figure 2:**
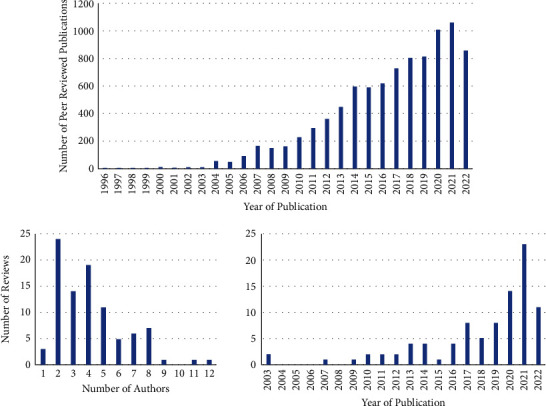
The number of BCI-reviewed publications after 1996 by year, and the number of review publications selected for this study showing an increasing trend to publish reviews and the distribution of the number of authors on those selected reviews.

The number of publications in the field of BCI has increased through time, commencing with two in 1972, one in 1988, and a constant gradual increase from 1996 (Figure 2). Similarly, there was an increase in the annual rate of review publications between 2003 and 2022 (Figure 2). The average number of authors in a review was 4.2 ± 1.8, the largest number of authors on any one review being twelve, with only three reviews having one author (Figure 2).

**Figure 3:**
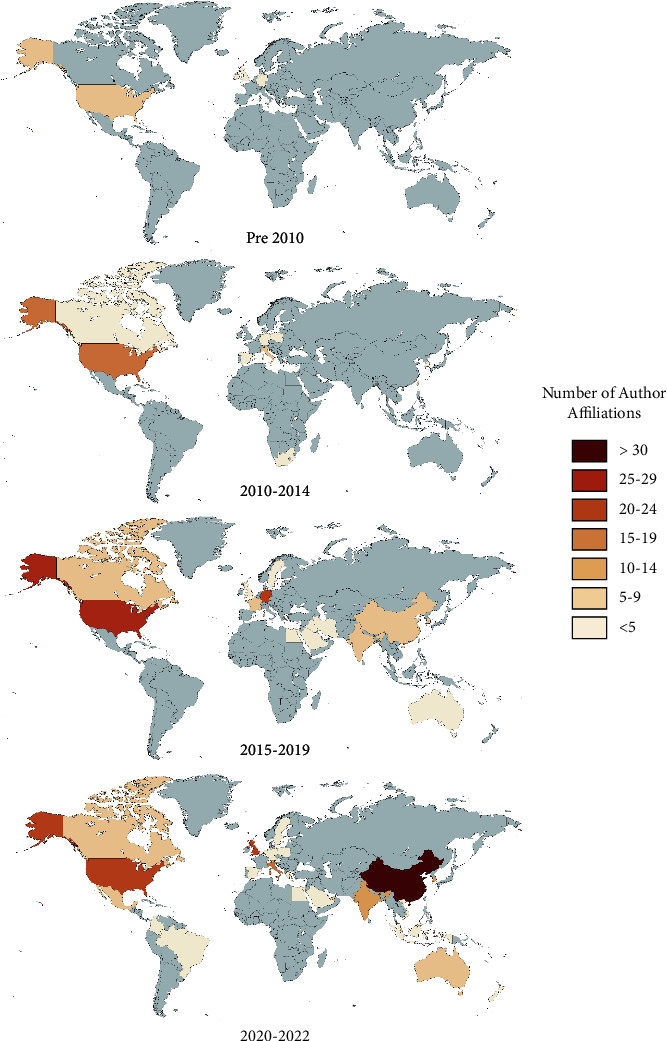
A map of review author affiliations showing changes in quinquennial blocks demonstrating a geographical shift from Euro-American dominated research to being more globally inclusive.

There has been a shift from a Euro-American dominance in author-affiliated institutions of BCI review writers to a more global domain, and currently, China dominates the author affiliation list (Figure 3). Before 2010, four countries contributed reviews, and these were written by authors with affiliations in Europe and the United States where authors were the most prolific publishers (*n* = 8; Figure 3). During the period 2010–2014, a total of 11 countries had authors affiliated with their institutions; these were dominated by the Europeans and the United States which had the highest number of author affiliations (*n* = 19). This period also saw one South African-affiliated author, seven from Taiwan, and five from South Korea showing sift from the western dominance in reviews. Further expansion of country author institutional affiliation occurred between 2015 and 2019, with 14 countries represented in the review literature. The United States (*n* = 28) and Germany (22) had the highest number of affiliated authors, with China (*n* = 9), India (*n* = 9), and South Korea (*n* = 6) forming the second tier of countries outside Europe and the United States. By 2020–2022 a total of 28 countries had authors with institutional affiliations. This period saw China rise to the top of the author affiliation list (*n* = 64), well ahead of the United States (*n* = 24), the United Kingdom (*n* = 23), and Italy (*n* = 15). Again, India (*n* = 10) and South Korea (*n* = 15) were prominent in author affiliations.

**Figure 4:**
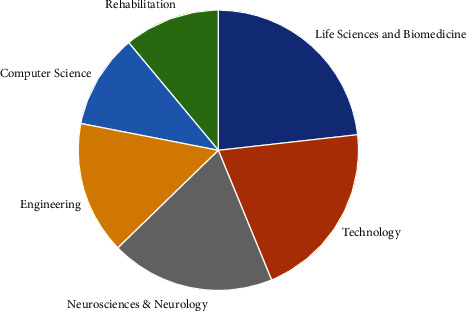
The disciplines identified in the *One Search* subject filter from the BCI review search show the relative importance of each discipline in proportion to each other based on the search metrics.

The *One Search-Subject Filter* indicated that there were six disciplines related to the review search (Figure 4). There were two overarching domains: (1) the first domain related to the theme of medicine: life sciences and biomedicine (*n* = 42), neurosciences and neurology (*n* = 35), and rehabilitation (*n* = 20) and (2) the second domain centered on the theme of functionality: computer science (*n* = 20), engineering (*n* = 28) and technology (*n* = 38).

### 4.2. Thematic Analysis

**Figure 5:**
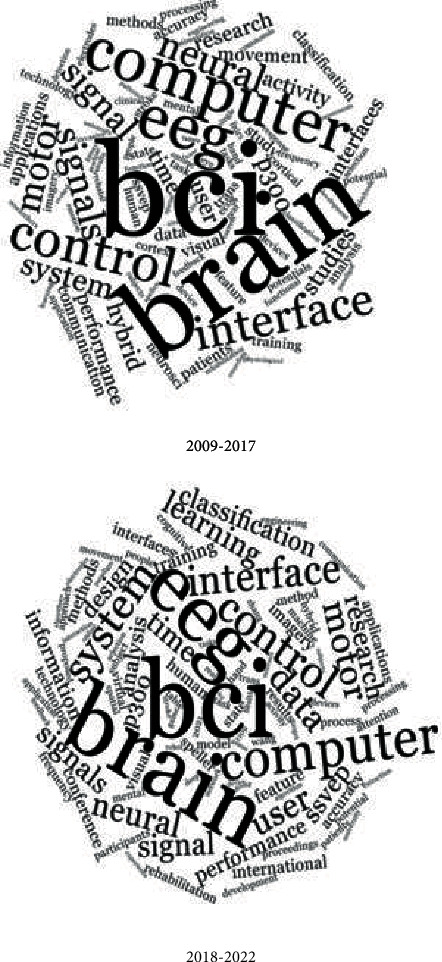
The word cloud generated to show word frequency use in BCI reviews for the period before 2018 and is contrasted with 2018 onwards.

The word clouds reveal that the keywords “BCI,” “brain,” and “computer” dominate. The terms “EEG” became in more frequent use in the reviewed literature from 2018, and “control,” “interface,” and “system” formed the main keyword for both periods (Figure 5). The change in research focus was also evidenced through the shift in language use, with terms such as “speller” appearing in reviews after 2018 [87–89]. Other words that had a greater frequency of use in the later cloud were “classification,” “data,” “design,” “information,” and “learning” which are closely associated with refining BIC systems, and the associated growth with BCI, AI and DL [10–12, 14–18], all of which appear in reviews post 2018.

The term “hybrid” was a word that became less frequently used in 2018. A title search of “hybrid” indicates that the topic was mostly reviewed between 2016 and 2017, with three review papers examined containing “hybrid” in their titles from 2018 onwards [14, 44, 60–63, 65] (Figure 5).

The word cloud and word cluster analysis are informative for what they do not exhibit. There is a lack of ethics and risk-benefit-associated terms. Consent, identity, humanity, liability, personal, responsibility, and stigma are absent, although these are major ethical themes concerning the use of BCI systems [22]. Similarly, words associated with the level of control an individual has in relation to the synchronous systems were not themes identified in the word frequency analysis. Much of the ethical debate on BCI research revolves around informed consent, privacy, risk, and security of the individual; again, these were lacking in any form in both the word cloud and its associated word cluster analysis [73, 90, 94]. It can be argued that ethical issues are not seen as structurally important, rather based on the lack of thematic detection, may be said to only extend to the adherence to the conditions surrounding institutional approvals that pertain to specific projects. Other than where the individual has the capability to provide levels of informed consent, there is a distinct gap in the review literature on the ethical issues that surround BCI system applications, and up until 2017, only 42 research publications contained a meaningful discussion on BCI ethical issues [22]. The lack of ethical terms in the word cloud and its associated world cluster analysis, particularly with the growing use of human applications that this analysis revealed, shows there is a need for ethics to be given greater consideration.

**Figure 6:**
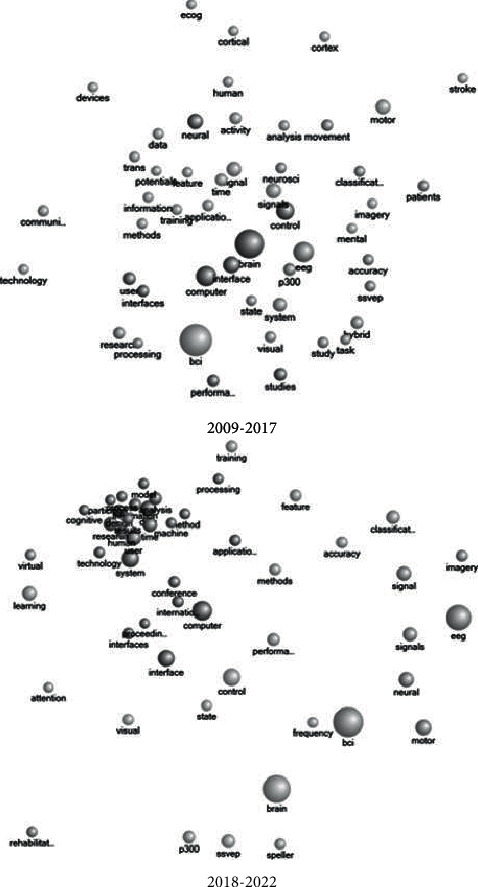
A word-association frequency diagram constructed from the BCI review publications using the Pearson correlation coefficients showing the contrasts in word association between pre-2018 and 2018 onward.

The center of the word-association frequency diagram illustrated the away from “BCI” and its composite words to “control,” “performance” and “state,” highlighting the shift in the review literature focus away from how BCI system operates and the move to the practical real-world application [4, 26, 27, 29, 35, 37, 90] (Figure 6). There is a district clustering in the 2018 onward diagram that is indicative of the focus center of research and is centered on “human,” “user,” and terms such as “cognitive” “process,” “technology,” and “response,” terms associated with the application of BCI [29, 40, 44, 97] (Figure 6).

Early reviews focused on the methods and achieved greater performance with words such as “access pathways” [66], “alternative communication” [67], “biocompatibility” [43], “classification” [57], “cognitive tasks” [31], “control” [33], “motor imagery” [7, 36, 70, 79], “neuroengineering” [19, 43] “neuroimaging” [37], and “sensory-motor regions” [79], all of which show a focus on improving the understanding of brain function and how BCI systems are able to exploit this knowledge.

This shift from understanding the brain function and how BCI systems could be used to a more nuanced and applied approach is reflected in the shift in keywords authors used. Some keywords remained in constant use highlighting that there are key themes of research across time periods. These terms include “signal possessing” or “signal analysis” [6, 7, 19, 52]. Medical-associated words such as “stroke” and “rehabilitation” were seen to remain in use [64, 71, 76, 78, 85]; this is reflected in the shift of focus from understanding how the brain functions after a stroke to more practical applications identifying functional brain pathways using“motor imagery” [7, 36, 70, 79, 89] and its practical application with “upper limb” [84, 85], or “upper extremity” [86] and “lower limb” [84].

Interestingly, 2017 saw a blush of terms associated with software and hardware with keyword terms such as “affective computing” [54], “BCI hardware” [4, 5], “BCI software” [4, 5], “classification accuracy” [61], “covariance matrix” [6], and “Riemannian geometry” [6]. In contrast, the 2018 onward period saw a shift toward technological development to exploit the early research with terms such as “learning” [55, 97], “robotics” [83], and “speller” [87].

## 5. Conclusion

This review highlights the shift through time in research themes from understanding brain faction and how BCI systems could use varying signal pathways to achieve effective BCI control of systems to more applied BCI research. Similarly, while there remains a focus on medical applications of BCI systems, there is an increasing interest in the commercialization of BCI systems for education, entrainment, gaming, marketing, and security proposes and this is reflected in the review topics.

Medial applications still dominate BCI research themes. Early research focused on the development applications, signal questions, system optimization, and particularly for theses surrounding control of an area of diagnostics and for rehabilitation. Achieving this has meant continued seeking of an understanding of how damaged areas of the brain are bypassed. Later medical research sought to build on the increased understanding of brain function and BCI systems with the development of practical applications for those with cognitive impairment of movement and function control.

Other themes in the research indicate a shift away from pure medical applications. This has occurred as BCI systems become more accurate, portable, less invasive, and more user friendly. The review literature points to BCI system applications that enhance the human experience in terms of augmenting the gaming and entertainment systems. Similarly, marketers and advertisers are using emotional detection BCI systems to improve advertising and marketing material to maximize a desired emotional response. Safety and security system development is focused on road safety applications and how BCI systems can be used in smart cities to control buildings and other infrastructure systems. The military is studying BCI systems to provide real-time information on the state of soldiers that are deployed [98].

### 5.1. Future Research

As further research advances in BCI systems are commercialized, the thematic trend is a refocusing away from medical application to a more “normalized” application in the lives of everyday individuals. This shift raises ethical questions of control, systemic societal behavior monitoring, and what level of oversight is in place not only for those that operate BCI systems but also those who gather the information that is collected using them. Ethics remain one of the major areas where there is a significant need for greater and more transparent oversight, and therefore, more research into ethics is a major priority for future research. It is expected that future themes will reflect the shift toward BCI systems becoming invasive into the lives of “normal” people, and it is expected that this thematic shift will also come with an increased call for research into ethical issues and the need for legal oversight.

## Data Availability

The data supporting this study's findings are available from the corresponding author upon reasonable request.
